# Culinary Herbs and Spices: Their Bioactive Properties, the Contribution of Polyphenols and the Challenges in Deducing Their True Health Benefits

**DOI:** 10.3390/ijms151019183

**Published:** 2014-10-22

**Authors:** Elizabeth I. Opara, Magali Chohan

**Affiliations:** 1 School of Life Sciences, Kingston University, Penrhyn Road, Kingston upon Thames KT1 2EE, UK; 2 School of Sport, Health and Applied Science, St. Mary’s University, Waldegrave Road, Strawberry Hill, Twickenham TW1 4SX, UK; E-Mail: Magali.chohan@smuc.ac.uk

**Keywords:** herbs, spices, polyphenols, bioactive properties, health

## Abstract

Herbs and spices have been used for both culinary and medicinal purposes for centuries. Over the last decade, research into their role as contributors of dietary polyphenols, known to possess a number of properties associated with reducing the risk of developing chronic non-communicable diseases, has increased. However, bearing in mind how these foods are consumed, normally in small quantities and in combination with other foods, it is unclear what their true benefit is from a health perspective. The aim of this review is to use the literature to discuss how preparative and digestive processes, bioavailability and interactions between foods may influence the bioactive properties of these foods, and whether or not polyphenols are responsible for these properties. Furthermore, this review aims to highlight the challenges that need to be addressed so as to determine the true benefits of these foods and the mechanisms of action that underpin their purported efficacy.

## 1. Introduction

The leaf, root, bark, berry, bud, seed, stigma of a plant or flower used for the purpose of cooking are commonly referred to as herbs and spices, which were and are primarily used for, and associated with, adding to or enhancing the flavor of foods including meats, sauces, vegetables and desserts [1]. Beyond acting as a replacement for salt [2], the nutritional contribution of these dietary plants has in the past been deemed negligible probably because of the relatively small, although increasing amounts consumed [3,4]. However, the literature indicates that within the last decade, this view is beginning to change [1].

The long historical use of herbs and spices for their medicinal benefits is fully acknowledged, and there is a growing amount of literature concerning the potential/purported benefits of these foods from a health perspective. These benefits include their possible role in conferring protection against cardiovascular and neurodegenerative diseases, cardiovascular disease, cancer and type 2 diabetes [1,5,6,7,8,9]. However, what is unclear is the true significance of these “benefits”. In their comprehensive review on the health benefits of herbs and spices Tapsell *et al*., [1] argue that the real challenge in establishing the role of these foods in maintaining health is not proving that they “have health benefits but in defining what these benefits are and developing methods to expose them by scientific means”.

One approach that is being used to begin to address this challenge is to investigate the bioactive properties of these foods within a nutritional context (that is investigating whether or not such properties are evident at levels at which herbs and spices are consumed). This approach has led to questions about the contribution of a group of phytochemical constituents which predominate in herbs and spices—the polyphenols [10,11,12,13]—and ultimately whether or not herbs and spices have a meaningful “health” role to play via their contribution to dietary polyphenol intake.

## 2. Dietary Polyphenols in Culinary Herbs and Spices

Polyphenols are a large family of structurally diverse compounds that can be classified based on the number of phenol rings (hydroxyl groups attached to aromatic rings) and the groups that bind these rings [14,15,16]. The main classes/groups of dietary polyphenols are the phenolic acids (hydroxybenzoic and hydroxycinnamic acids), the flavonoids (flavonols, flavones, isoflavones, flavonones, flavanols and the anthocyanidins), the stilbenes and the lignans. Other classes of dietary polyphenols are the coumarins and the tannins: the coumarins are cinnamic derived phenolic compounds and the term tannin is applied to large polyphenolic compounds including proanthocyanidins and gallic acid esters–moleular weight > 500) [15,17,18,19]. A large number of polyphenols occur in nature however environmental factors, both biotic and abiotic, affect the polyphenolic content of foods. Furthermore, the type of diet may limit the level and composition of intake [11,12,13,15,16,20].

Polyphenols are found in numerous plant derived foods including herbs and spices, which, especially in their dried forms, generally contain relatively high levels of polyphenols compared to other polyphenol rich foods including broccoli, dark chocolate, red, blue and purple berries, grape and onion [11,12,13,21] (Table 1 and Table 2).

**Table 1:** Total phenolic content of common culinary herbs and spices.

Food			Total Phenolic Content (mg/100 g FW ^a^) ^b^
Herbs	Coriander (*Coriandrum sativum* L.)	dried	2260
		fresh	158.90
	Dill (*Anethum graveolens* L.)	dried	1250
		fresh	208.18
	Oregano (Wild Majoram) (*Origanum vulgare* L.)	dried	6367
		fresh	935.34
	Parsley (*Petroselinum crispum* (P. Mill.))	dried	1584
		fresh	89.27
	Rosemary (*Rosmarinus officinalis* L.)	dried	2518
		fresh	1082.43
	Sage (Common) (*Salvia officinalis* L.)	dried	2919
		fresh	185.20
	Thyme (Common) (*Thymus vulgaris* L.)	dried	1815
		fresh	1173.28
Spices	Cinnamon (Ceylan) (*Cinnamomum verum* J. Presl)		9700
	Cloves (*Syzygium aromaticum*)		16,047.25
	Coriander seed (*Coriandrum sativum* L.)		357.36
	Ginger (*Zingiber officinale* Roscoe)	dried	473.50
		fresh	204.66
	Nutmeg (*Myristica fragans* Houtt.)		1905
	Turmeric (*Curcuma longa* L.)		2117

^a^ FW: Fresh weight; and ^b^ values obtained from Phenol-Explorer Database Neveu *et al.* [11], Pérez-Jiménez *et al*. [12], Pérez-Jiménez *et al.* [13].

**Table 2:** Total phenolic content of other polyphenol rich foods.

		Total Phenolic Content (mg/100 g FW ^a^) ^b^
Food	Dark Chocolate	1859.80
	Broccoli (*Brassica oleracea* var. *italica Plenck)*	198.55
	Blackcurrant (raw) (*Ribes nigrum* L.)	820.64
	Red raspberry (raw) (*Rubus idaeus* L.)	148.10
	Strawberry (raw) (*Fragaria* L.)	289.20
Blueberries	Half Highbush (*Vaccinium augustifolium* Ait. × *Vaccinium corymbosum* L.)	151.33
	Highbush (raw) (*Vaccinium corymbosum* L. and *Vaccinium corymbosum* L.)	223.4
	Lowbush (raw) (*Vaccinium augustifolium* Aiton)	471.55
	Rabbiteye (*Vaccinium corymbosum* L.)	549.98
Cranberry	American (*Vaccinium macrocarpon* Ait.)	315.00
	European (*Vaccinium oxycoccos* L.)	139.50
Grape (*Vitis vinifera* L.)	Black	184.97
	Green	121.80
Onion (raw)	Red (*Allium cepa* var. *cepa* L.)	102.83

^a^ FW: Fresh weight; and ^b^ values obtained from Phenol-Explorer Database Neveu *et al.* [11], Pérez-Jiménez *et al.* [12], Pérez-Jiménez *et al.* [13].

The predominant class/group of polyphenols in herbs and spices are the phenolic acids and flavonoids (mainly the flavone and flavonol sub groups). However, some, (parsley (*Petroselinum crispum*), Chinese cinnamon (*Cinnamomum aromaticum* Nees) ginger (*Zingiber officinale*), turmeric (*Curcuma longa* L.)) also contain other sub-groups of polyphenols (furanocoumarins (parsley), hydroxycoumarins (Chinese cinnamon), hydoxyphenylpropenes (ginger), curcuminoids (turmeric)) [11,12,13,22,23,24,25,26,27,28,29,30,31,32,33,34,35,36,37,38,39].

### Properties of Polyphenols

Polyphenols and polyphenol rich foods especially fruits, vegetables and green tea, are widely known for their antioxidant properties however they exert other biological effects (anti-inflammatory, anti-cancer and neuro-protective), which may also contribute to their purported benefits, possibly or not, via their antioxidant properties, and they are therefore linked to the maintenance of health via protection against the development of non-communicable diseases [16,19,40,41,42,43,44,45,46,47,48]. Other properties include anti-microbial, anti-diabetic (Type II), and anti-asthma activities [16,49,50] and there is now a growing amount of literature on how polyphenols confer health benefits via their action on gut microbiota [51,52,53,54].

Culinary herbs and spices have also been shown to possess these properties [1,5,7,8,10,55,56,57,58,59,60,61,62,63,64,65,66,67,68,69,70]. Furthermore, evidence suggests that it is the polyphenols that have a significant role to play in conferring these properties [1,10,57,58,70,71] and as these foods have high polyphenol contents they may be important dietary sources of the purported protective properties that their polyphenols confer [57,58,64,70]. In order to begin to determine if culinary herbs and spices are indeed significant dietary contributors of polyphenols, and their properties, the following need to be considered: Habitual levels of intake; the significance of their bioactive properties at habitual levels of intake; how the preparative and digestive processes they undergo prior to, and after consumption, respectively, and absorption affect these properties (especially as much of the research on the bioactive properties of these foods has been done on their uncooked state); and the influence of other foods on the bioactive properties of herbs and spices (as they are rarely consumed on their own).

## 3. Habitual Levels of Intake of Culinary Herbs and Spices

In light of the bioactive properties that culinary herbs and spices possess, the need to determine their intake is being acknowledged. Intake data are available for particular groups/populations [3,4,72] (Table 3). The levels of intake are clearly and predictably much lower than for foods more widely known for their protective properties, and the values provided vary considerably possibly due to a number of factors that are very difficult to control including, under-reporting, the inclusion of non-herb and spice seasonings, the large varieties of recipes for a given dish, how such recipes are interpreted, appetite and portion size [3,4,70,72]. However, the relatively low intake levels of culinary herbs and spices do not necessarily mean that they are of little value as their high polyphenol content, and thus ultimately the potential biological impact of this content, cannot be ignored.

**Table 3:** Culinary herb and spice intake studies.

Study	Intake Data
Pellegrini *et al.* [72]: Determined daily intake of spices using 3 day weighed food record (3D-WR) and food frequency questionnaire (FFQ). For the 3D-WR median data were obtained. For the FFQ, interquartile range data were obtained. *n* = 285; Subjects: men (*n* = 159) and women (*n* = 126); Country of study: Italy	0.4 (1.3) g (3D-WR); 3.2 (2.7) g (FFQ)
Carlsen *et al.* [3]: Determined herb and spice intake using a FFQ and 2–4 weeks later 28 days recording of herb and spice consumption (HSR). *n* = 146; Subjects: men (*n* = 63) and women (*n* = 83); Country of study: Norway	Median estimates of total herb and spice consumption: 2.7 g/person/day (range 0.19–45.0; Interquartile range 4.4) from the FFQ; 1.6 g/person/day (range 0–10; interquartile range 1.8) from the HSR; Main herb/spice contributors: Basil (dried and fresh), oregano (dried), cinnamon, pepper, and spice blends
Pérez-Jiménez *et al.* [4]: Measured the contribution of seasonings (included non-herb and spice seasonings) to daily polyphenol intake using 24 h dietary records every 2 months from 1995–1996 and the Phenol-Explorer database. Mean intake data obtained. *n* = 4942; Subjects: men (*n* = 2596) and women (*n* = 2346); Country of study: France	0.4 (0.3) mg/day/person; Main herb/spice contributors: Ginger and parsley

## 4. The Impact of Processes Prior to, and Post Consumption on the Bioactive Properties of Culinary Herbs and Spices

### 4.1. Antioxidant Capacity and Total Phenolic Content

Culinary herbs and spices are, in many instances, cooked prior to consumption, and then undergo digestion. Thus, to further elucidate their biological and nutritional significance, the impact of these processes needs to be understood especially at levels of habitual intake. Chohan *et al*., [20] carried out a study on the impact of cooking on a number of common culinary herbs and spices, namely cinnamon, cloves, fennel, ginger, parsley, rosemary, sage and thyme at amounts used in the preparation of food (0.2–1 g). Microwaving, simmering and stewing all increased the antioxidant capacity probably as a result of heat liberating the antioxidant compounds [73,74]. In contrast, cooking techniques that involved dry heating, grilling and frying, resulted in a decrease in antioxidant capacity which was associated with browning and thus may be indicative of the Maillard reaction, or more specifically its products, influencing antioxidant capacity [74,75,76].

Subsequent studies using culinary herbs from the Lamiaceae family: parsley, rosemary, sage and thyme showed that changes in antioxidant capacity due to cooking processes were strongly and significantly associated with changes in total phenolic content [72,77]. This finding is not surprising as the assays used to determine antioxidant capacity (Trolox equivalent antioxidant capacity/2,2'-Azinobis-(3-ethyl benzothiazoline-6-sulfonic acid) assay-TEAC/ABTS assay) and total phenolic content (the Folin-Ciocalteau/gallic acid equivalents–GAE-assay) are based on similar redox reactions [78] however the role of polyphenols as major contributors of antioxidant capacity in culinary herbs and spices is well established [10,32,57,79,80]. Chohan *et al*., [71] also showed that the effect of cooking on antioxidant capacity is not always consistent, which is an observation that may be associated with the nature of the food matrix and the type of cooking method used [81,82,83]. Related work by Chohan [77] also showed that cooking time can also affects the antioxidant capacity (TEAC), total phenolic content (GAE) and polyphenol, specifically phenolic acid, profile of culinary herbs.

Chohan *et al*., [71] also investigated the impact of digestion post cooking on the antioxidant capacity and total phenolic content of these culinary herbs and found that both were significantly increased compared to uncooked and also cooked culinary herbs. Other studies on the impact of digestion, *in vitro*, on antioxidant capacity/activity and/or total phenolic content reported a decrease in fruit beverages [84], either no change or a decrease for herbal teas, prepared from infusions of powdered herbs [85] or either decreases, increases or no statistically significant change for dietary antioxidant supplements [86]. The type of *in vitro* model (*i.e.*, the chemical and enzymatic environment within the models) used could account for some of these differences [87]. However the nature of the food/delivery matrix may have also influenced the outcomes of these studies and thus suggests that the form of the food may contribute to its impact as a dietary contributor of the bioactive properties of its constituents.

Preliminary HPLC analysis of rosemary, sage and thyme (uncooked, cooked and cooked and digested) strongly suggest that significant increases in rosmarinic acid, which is a predominant phenolic acid in these herbs, is most likely responsible for the observed increases in antioxidant capacity following cooking and also cooking and digestion. These results also suggest that to some extent other phenolic acids including caffeic acid, ferulic acid and vanillic acid may also contribute to the increase reported for some but not all of the herbs investigated [77]. These results certainly strengthen the role of polyphenols in the conferment of antioxidant properties on to these foods. They also highlight the variations that occur in the chemical composition of different batches of foods as there was only partial agreement with data obtained from the Phenol-Explorer database [11,12,13] specifically for the uncooked herbs. These variations could be due to biotic and abiotic factors as stated above [57,88] and/or the sensitivity of the analytical technique used.

For culinary spices, Baker *et al*., [70] found that cooking and digestion affected the antioxidant capacity and total phenolic content of cinnamon, cloves and nutmeg-again at levels associated with the preparation of food. However, these changes were not consistent. One possible reason given for this is the behavior of the phytochemical constituents (both polyphenol and non-polyphenolic) within the food matrix during these processes giving rise to additive, antagonistic and/or synergistic effects on the bioactivity of these foods.

### 4.2. Anti-Inflammatory Activity and Total Phenolic Content

There is a paucity of data concerning the effect of preparative and digestive processes on the anti-inflammatory activity of culinary herbs and spices. Investigations of the impact of cooking and digestion on the anti-inflammatory properties of culinary herbs and spices demonstrate that this property is not diminished by these processes. Chohan *et al*., [71] reported that at amounts used in food preparation, uncooked, cooked, and cooked and digested rosemary, sage and thyme elicited an anti-inflammatory effect via the inhibition of, and also protection against, the action of pro-inflammatory agents hydrogen peroxide (H_2_O_2_) and tumor necrosis factor α (TNFα) which resulted in the inhibition of IL-8 release from peripheral blood lymphocytes (PBLs). These decreases were only significant for PBLs exposed to H_2_O_2_ for the most part which may be indicative of an activity that involves more than the inhibition of a single pro-inflammatory mediator. There was a strong and significant correlation between inhibition of IL-8 release and antioxidant capacity and total phenolic content irrespective of whether the herbs were uncooked, cooked or cooked and digested which indicates that the polyphenols within these foods contribute to this anti-inflammatory activity, and that this activity may be due to their antioxidant properties. However, the findings of Baker *et al*., [70] which were focused on culinary spices at levels associated with habitual intake suggest that the contributory role of polyphenols is not so straight forward (as indicated by Chohan *et al.* [71]): Baker *et al*., [70] reported that the spices cinnamon, clove and nutmeg (uncooked, cooked and digested) significantly inhibited the pro-inflammatory enzyme cyclo-oxygenase-2 (COX-2). The study also reported via correlation analysis that the anti-COX-2 activity was only partially associated with the antioxidant capacities and polyphenolic content of these spices. The partial correlation to phenolic content suggests the involvement of non-polyphenolic compounds, which is supported by the literature. For example, cinnamaldehyde, a major constituent of cinnamon, and the essential oil responsible for its aroma and flavor [89], has been shown to inhibit COX-2 activity [90]. The partial correlation with antioxidant capacity suggests that other actions may contribute to the anti-inflammatory properties.

The inflammatory response is a complex one and involves numerous mediators, a number of which may be affected by individual polyphenols and thus by culinary herbs and spices: Yoon and Baek [91] discuss the inhibitory effect of polyphenolic compounds including phenolic acids and flavonoids on one or possibly several cellular pathways that are involved in the inflammatory process. These pathways include the arachidonic dependent pathways, which involve the action of the cyclo-oxygenase (COX) enzymes, and the arachidonic independent, pathways, which include peroxisome proliferator activated receptors (PPARs), nitric oxide synthase (NOS), nuclear transcription factor κB (NF-κB), which regulates the expression of pro-inflammatory cytokines including IL-8, as well as the non-steroidal anti-inflammatory drug (NSAID) activated gene. Some of these polyphenols include those that are found in culinary herbs and spices. For example, rosmarinic acid has been shown to inhibit the pro-inflammatory PKC/NF-κB pathway [92]. Curcumin, a predominant polyphenol in turmeric, also inhibits NF-κB [93] COX-2 has also been shown to be down-regulated and/or inhibited by eugenol (clove) and apigenin (parsley) [94,95].

Culinary herbs, including lemon grass, rosemary and thyme are also reported to enhance the activity of the enzyme superoxide dismutase (SOD), which in addition to being an important antioxidant enzyme has the potential to act as an anti-inflammatory agent as it catalyses the dismutation of the free radical superoxide, which is associated with chronic inflammation [96,97]. A recent study by Chohan *et al.* [98] identified rosemary, sage and thyme as possessing superoxide dismutase mimetic (SODm) activity which was significantly associated with the antioxidant capacity, total phenolic content and inhibition of IL-8 release. The association with the former (antioxidant capacity) is not surprising but this analysis does indicate that polyphenols in these herbs may be responsible for the SODm activity as suggested by Huaefi and Smetanska [99] and that it (SODm) may also contribute to the herbs’ anti-inflammatory activity. Thus, this mimetic activity possessed by these herbs may account for some of the antioxidant and anti-inflammatory properties that these foods possess.

In summary, studies on the antioxidant and anti-inflammatory activities of culinary herbs and spices at levels associated with habitual intake show that these properties are not diminished post cooking and digestion. In addition, this work also indicates, via correlation analysis, that polyphenols are significant in conferring both these activities. However, for some spices non-polyphenolic compounds may also have a role to play.

## 5. Bioavailability of Polyphenols from Culinary Herbs and Spices

For a clearer understanding of the significance of the potential health benefits of culinary herbs and spices, it is essential to establish the bioavailability of their bioactive constituents. The literature on the bioavailability of polyphenols reports that the intestinal absorption of dietary polyphenols into systemic circulation is poor as they are metabolized by gut flora and/or the liver (post-absorption), and/or eliminated from the body rapidly [15,17,100]. Ultimately these factors will influence their activity and thus their health effects significantly. A preliminary study by Chohan *et al.* [101], carried out using a Caco-2 *in vitro* model, which has a high level of agreement with bioavailability studies in humans [102,103], on the bioavailability of polyphenols from culinary herbs (1 g starting amounts, post cooking and digestion) found that 8.3%–10.6% of the total phenolic content of cooked and digested rosemary, sage and thyme was detected post-passage through the Caco-2 monolayer. In addition, low levels of antioxidant capacity were also detected (8.5%–15% of pre-passage through the monolayer). Furthermore, despite the detection of polyphenolic content and antioxidant capacity following passage through the monolayer, no constituent polyphenols, specifically phenolic acids, were detected using HPLC. This finding at face value appears to contrast with that of Lee *et al*. [104] who reported detecting hydroxycinniamic acids post passage across a Caco-2 monolayer, although permeability across the monolayer was low. However, the lack of detection in the study by Chohan *et al*. [101] may be due to the small starting amounts of herb being diluted first during the cooking and *in vitro* digestion stage and then during the bioavailability experiments. The small starting amount was used to help establish the significance of the health promoting properties of the herbs *in vivo*, at levels that are used in the preparation of food, and are thus consumed. The samples used by Lee *et al*. [104] to investigate bioavailability were not foods but pure polyphenols, which did not undergo cooking and/or digestion prior to passage across the Caco-2 monolayer.

The observation regarding low bioavailability suggests that the action of dietary polyphenols may be localized to the gut, although how polyphenols interact with the gut is unclear [17,100]. In addition, there is a growing amount of literature on the positive role of polyphenols and polyphenol rich foods on gut health specifically with regards to the prevention and treatment of colorectal cancer [84,105,106,107,108,109,110,111,112,113,114,115] However, there are a number of additional factors that need to be considered when attempting to elucidate the action of the gut on these foods and their polyphenols. First, in the study by Chohan *et al*. [101] the time periods for the Caco-2 study were 60 and 120 min but it needs to be borne in mind that some polyphenols may remain in the small intestine for longer periods. In addition, the role of intestinal microflora also needs to be taken into consideration as they may be of potential significance with regards to making polyphenols more available for intestinal absorption [84]. The role of the colon needs to be factored in as the non-starch polysaccharide constituents, mainly cellulose, of the herbs investigated, although not affected in the gastric and small intestinal stages of digestion, could be metabolized by intestinal colonic microflora [17,100]. Thus, following cellulose breakdown, the polyphenols in these herbs could be metabolized in the colon. This theory is supported by a study which found large amounts of metabolites of phenolic compounds in human fecal water [116]. Furthermore, a study by Dall’Asta * et al*. [117] showed evidence of metabolism of dietary polyphenols following colonic fermentation, *in vitro*.

Research to establish the bioavailability of polyphenols in culinary herbs and spices highlights a plethora of factors that paradoxically contribute to and also limit our knowledge and understanding of the bioavailability of these compounds from these foods, the impact on their bioactive properties and ultimately their health benefits. To obtain further insight regarding the significance of bioavailability, studies on the impact of the food matrix and interactions between its constituents, the chemical, enzymatic and bacterial environment within the gut, and habitual, as opposed to single dose, intake are required [100,118,119].

## 6. Bioactive Properties of Combinations of Culinary Herbs and Spices: The Role of Synergy

It is fully acknowledged and recognized that identifying the constituents within foods that confer bioactive properties is important especially from a mechanistic perspective [18,120,121,122]. However, it is the whole food that is consumed, and it is normally consumed in combination with other foods, so a key question is: How do the food matrix and its constituents influence the whole foods’ bioactive properties? Answering such a question will facilitate the unravelling of the true health benefits of plant derived foods like culinary herbs and spices *in vivo* as they are commonly consumed in combination with each other and with other types of foods. There is a growing amount of literature regarding the efficacy of combinations of individual polyphenols, culinary and medicinal herbs, foods rich in polyphenols, and polyphenols and other protective phytochemicals based on their anti-proliferative and antioxidant properties *in vitro* (predominantly) and *in vivo*. De Kok *et al*., [123] provided a comprehensive review of the literature in this area in 2008 and recent studies subsequent to this work further highlight the importance of investigating the efficacy of these combinations [124,125,126,127,128,129,130,131,132] (Table 4). The findings of these studies indicate that the outcome of combining dietary polyphenols and their foods is influenced by the constituents themselves, the number of constituents (food or polyphenol) that make up the combination, the amount/concentration of a constituent, any processing that the combinations may undergo, for example cooking, and also the assay used [129,130,131,132]. Furthermore, the analysis used to determine if antagonism or synergy has occurred is another factor that needs to be considered [133]. Some studies use analysis based on the summation of effects method, which compares the effect of constituents combined with that of the expected effect, which is the sum of the effects of the individual constituents. However, it is argued that this method is limited when it comes to complex mixtures (such as food) as it depends on the mechanism of action of each constituent and assumes that the response of each constituent is linear in nature. Thus, another method, the isobole method, is more appropriate as it is independent of the mechanism of action and is said to apply under most conditions [133]. This method is more complicated as the different combinations used for the isobolographic analysis must have generated an iso-effect however it has been used to investigate interactions between herbs based on antioxidant and antiproliferative activities [125,129,131]. In summary, and as with bioavailability, studies on food synergy highlight the challenges of determining the benefit *in vivo* of culinary herbs and spices as well as fully elucidating the mechanisms that underpin their true efficacy.

**Table 4:** Recent studies on the antagonistic and synergistic effects of combinations of individual polyphenols or combinations containing polyphenol rich foods.

Combinations	Effect	Study
Epigallocatechin gallate (EGCG) and curcumin	Synergistically cytotoxic to MDA-MB-231 estrogen receptor α (ERα) human breast cancer cells *in vitro* when compared to effects of the individual polyphenols.EGCG + curcumin also synergistically inhibited tumor growth within female athymic nude mice implanted with MDA-MB-231 estrogen receptor (ERα) human breast cancer cells compared to individual polyphenols. Proposed mechanism of action: Cell cycle arrest and decrease in the expression of vascular endothelial growth factor receptor in tumor may play a role.	Somers-Edgar *et al.* [124]
Curcumin and resveratrol	Synergistic inhibition of growth of p53 positive and p53 negative human colorectal cancer HCT116 cells *in vitro* when compared to effects of the individual polyphenols.Curcumin and resveratrol combination also synergistically inhibited tumor growth within severe combined immunodeficient female mice implanted with HCT-116 cells. Proposed mechanism of action: Decrease in proliferation and induction of apoptosis, decreased NF-κB activity, inhibition of activation of epidermal growth factor receptor.	Majumdar *et al.* [125]
Carnosic acid and curcumin	Combinations (at levels shown to be non-cytotoxic to normal human fibroblasts or human peripheral blood mononuclear cells) inhibited the growth of, and induced apoptosis in, HL-60 and KG-1a human acute myeloid leukemia cells. Proposed mechanism of action: Apoptosis associated with activation of caspases 8, 9 and 3 and Bid (a proapoptotic protein) which is a member of the Bcl family. No other Bcl proteins shown to be affected. No evidence that oxidative stress was involved.	Pesakhov *et al.* [126]
Chicken +/−herb and spice based marinating sauces	Marinating and cooking significantly decreased the antioxidant capacities of herb and spice marinating sauces.	Thomas *et al.* [127]
Antioxidant rich spice (black pepper, cloves, cinnamon, garlic powder, ginger, oregano, paprika and rosemary) added to hamburger meat	Significant reduction in malondialdehyde concentration (a biomarker of oxidative stress) in the spiced burger compared to that in the unspiced (control) burger. There was also a significant increase in plasma malondialdehyde concentration following consumption of the control burger. Following consumption of the spiced burger there was a “trend to decrease” in plasma malondialdehyde concentration. Urinary malondialdehyde concentration decreased by almost 50% in subjects that consumed the spiced burgers compared to those who consumed the control burgers.	Li *et al.* [128]
Combinations of *Aspalathus linearis* and *Malus domestica*, *Aspalathus linearis* and *Vaccinium*, *Myrtillus*, *Punica granatum* and *Malus domestica*	Combinations demonstrated additive or synergistic effects (based on antioxidant capacity) but these outcomes depended on the type of assay used.	Blasa *et al.* [129]
Polyphenol rich herbs oregano, ajowan (*Trachyspermum ammi*) and Indian borage (*Plectranthus amboinicus*)	Addition of oregano extract increased the radical scavenging activity of ajowan and Indian borage extracts.	Khanum *et al.* [130]
Peppermint, rosemary, sage, spearmint, thyme.	All herb extracts inhibited the growth of SW-480 human colorectal cancer cells. Combinations of these extracts herbs had additive, antagonistic and synergistic effects, which were based on the combinations and/or the concentrations of the herb extracts used in the combinations.	Yi and Wetzstein [131]
Blueberries, grapes, chocolate covered strawberries, and polyphenol rich fruit smoothies.	Significant synergy, based on antioxidant capacity, found in combinations of chocolate covered strawberries; reported either antagonism or synergy within the combinations of constituent polyphenols; the effect depended on the constituents, and their number, and also the antioxidant assay used.	Epps *et al.* [132]

## 7. Conclusions

Current research on the impact of preparative and digestive processes on the bioactive properties of culinary herbs and spices has shed some light on their potential benefits. However, further work is needed to fully understand if the low bioavailability of polyphenols from these foods really limits their health benefits. Furthermore, there is very little understanding of the impact of combining these foods on their bioactivity. Ultimately, it is the use of a combination of *in vivo* and *in vitro* methods that will determine the true health benefits of culinary herbs and spices and the contributory role of their constituent polyphenols.
